# Vitamin E and Multiple Health Outcomes: An Umbrella Review of Meta-Analyses

**DOI:** 10.3390/nu15153301

**Published:** 2023-07-25

**Authors:** Zheyu Xiong, Linhu Liu, Zhongyu Jian, Yucheng Ma, Hong Li, Xi Jin, Banghua Liao, Kunjie Wang

**Affiliations:** Department of Urology, Institute of Urology (Laboratory of Reconstructive Urology), West China Hospital, Sichuan University, Chengdu 610041, China; xiongzhemiao@icloud.com (Z.X.); huaxijasonliu@icloud.com (L.L.); jianzhongyu@wchscu.cn (Z.J.); yuchengma88@stu.scu.edu.cn (Y.M.); lihonghxhx@scu.edu.cn (H.L.); jancy_king@hotmail.com (X.J.)

**Keywords:** vitamin E, CVD, cancer, meta-analysis, umbrella review

## Abstract

The relationship between vitamin E intake or circulating α-tocopherol and various health outcomes is still debatable and uncertain. We conducted an umbrella review to identify the relationships between vitamin E intake or circulating tocopherol and health outcomes by merging and recalculating earlier meta-analyses. The connections that were found to be statistically significant were then classified into different evidence levels based on *p* values, between-study heterogeneity, prediction intervals, and small study effects. We finally included 32 eligible meta-analyses with four vitamin E sources and 64 unique health outcomes. Only the association between circulating α-tocopherol and wheeze or asthma in children was substantiated by consistent evidence. Suggestive evidence was suggested for seven results on endothelial function (supplemental vitamin E): serum C-reactive protein (CRP) concentrations (supplemental vitamin E), cervical cancer (dietary vitamin E), esophageal cancer (dietary vitamin E), cervical intraepithelial neoplasia (CIN, dietary vitamin E), pancreatic cancer (total vitamin E intake), and colorectal cancer (circulating α-tocopherol levels); all of these showed a protective effect consistent with the vitamin E source. In conclusion, our work has indicated that vitamin E is protective for several particular health outcomes. Further prospective studies are required when other factors that may contribute to bias are considered.

## 1. Introduction

Vitamin E, as one of the four fat-soluble vitamins, is an essential nutrient for the human body and is crucial to the health of the human body. As an antioxidant, it can protect polyunsaturated fatty acids in membranes from oxidation, regulate the production of reactive oxygen species and reactive nitrogen species, and modulate signal transduction [1].

It is used to describe eight kinds of plant sources of fat-soluble compounds (α-, β-, γ-, δ-tocopherols and α-, β-, γ-, δ-tocotrienols). However, only α-tocopherol meets the body’s requirement for vitamin E, based on the fact that it is the form preferentially retained by the body after vitamin E intake. α-tocopherol transfers a protein (α-TTP), expressed in the human liver, which is closely related to maintaining the circulating concentration of α-tocopherol; α-TTP protein is poor at recognizing the other seven forms of vitamin E [2]. Therefore, in the United States, the conclusion that only α-tocopherol meets the definition of vitamin E were supported by the Food and Drug Administration (FDA), which requires certain labeling of foods and supplements. 

Vitamin E is widely found in foods and fruits in our daily lives, especially oils, nuts, and seeds [3]. Therefore, patients, healthcare providers, and people in general are concerned about the relationship between vitamin E and human health. There is a significant and quickly growing body of literature that examines the relationships between vitamin E intake or circulating α-tocopherol levels and numerous disorders and illnesses. For example, according to some research, taking vitamin E supplements may help prevent cardiovascular disease (CVD) by acting as an antioxidant, preventing the oxidation of lipoproteins, and avoiding platelet aggregation [4]. However, some high-quality studies have not supported the prevention of CVD through vitamin E supplementation [5]. This discrepancy was also seen in research on the link between vitamin E and cancer [6]. In addition, epidemiological studies have indicated that vitamin E is also related to neurodegenerative diseases [7], age-related macular degeneration [8], non-alcoholic fatty liver, [9] and so on. 

Therefore, we conducted this umbrella review in order to collect, summarize, and assess the quality and strength of the available evidence for meta-analysis, providing an overall picture of vitamin E intake or circulating α-tocopherol with health outcomes and to establish high-quality evidence for therapeutic decisions.

## 2. Methods

Our protocol has been registered in PROSPERO (CRD42021292442).

### 2.1. Literature Search and Selection Criteria

We performed systematic searches in PubMed, EMBASE, and the Cochrane Library of Systematic Reviews to identify systematic reviews and meta-analyses that investigated the relationships between vitamin E intake or circulating α-tocopherol and various health outcomes up to November 2021. We used the following search strategy: (tocopherols OR terms related to vitamin E) AND (systematic review OR meta-analysis) using truncated terms for all fields (Table S1). Two authors conducted a literature search using the suggested search criteria for systematic reviews and meta-analyses in the SIGN guidance. Conflicts were settled through consensus. Additionally, we looked through the references of pertinent articles.

### 2.2. Selection of Meta-Analyses

Articles were eligible if they were meta-analyses and were conducted using a systematic approach. We included meta-analyses of observational (cohort, case-control, and cross-sectional studies) and randomized controlled trials (RCTs). We used the PRISMA flowchart to record the study selection process [10]. All eligible meta-analyses evaluated the relationship of vitamin E intake or circulating α-tocopherol with any health outcomes. Studies on genetic variants for Vitamin E metabolism were the exception, as they did not examine any health outcomes for which vitamin E had been explored as the exposure of interest (for example, vitamin E receptor).

Studies conducted in laboratories and on animals were not included. We included each meta-analysis that was included in an article if it had one for each of the several health outcomes. To prevent the inclusion of duplicate studies, we only selected one meta-analysis for each health outcome when multiple meta-analyses addressed the same research issue. In that instance, we included the one with the most primary research or the one that has been updated most recently. 

### 2.3. Data Extraction

The data was extracted by one author and was confirmed by another. We took note of the first author, publication year, vitamin E source, populations, number of studies, research design(s), funding details, and any conflicts of interest for each qualifying meta-analysis. We also extracted the risk ratio, odds ratio, mean differences, standardized mean differences, and weighted mean differences, as reported by the authors in the meta-analysis, and the corresponding 95% CI.

We also retrieved the Egger’s *p* value to assess publication bias, estimates of the fraction of variance representing real variations in impact size (*I*^2^), and any estimate of variance between trials (τ^2^) [11]. Any ambiguity or differences were resolved through discussion.

### 2.4. Methodological Quality Assessment

Each meta-analysis was evaluated for methodological quality using Online AMSTAR 2, a metric for assessing systematic reviews; it is a 16-item checklist that makes up the AMSTAR 2 quality evaluation instrument [12]. The validity of a review and its results can be significantly affected by seven crucial criteria in AMSTAR 2. It should be noted that the selection of key items can be adjusted according to specific circumstances. Two researchers rated the methodological quality. Any differences were resolved by consensus.

Using the GRADE methodology, the strength of the evidence for each outcome covered by the umbrella review was assessed [13]. There are eight domains in it that have the potential to raise or decrease the confidence of the evidence. Evidence from eligible meta-analysis was ultimately assigned into four categories ranging from high quality to very low quality.

### 2.5. Data Analysis

We recalculated the selected meta-analysis for each health outcome using the precise relative risk estimates. We updated the summary effect size and associated 95% Cl using DerSimonian and Laird’s random-effects model, which takes into account heterogeneity both within and between trials. The between-study heterogeneity was recalculated using the Q test *p* value and *I*^2^ statistic. We also estimated the 95% prediction interval (PI) using recalculated data. The distribution of true effects, represented by the 95% PI, is where 95% of original research on the same issue will fall [14]. And correlations between vitamin E and the risk of health outcomes were divided into 4 categories (Table S2).

### 2.6. Patient Involvement

The questionnaires and health outcome measures for this study were not developed with input from any patients. No patients were consulted on the umbrella review’s research design or how to interpret the findings. We are planning to engage local policy maker to disseminate the research through social media and publish the summary of the findings at the website of the Cochrane China Center.

## 3. Results

### 3.1. Characteristics of Meta-Analyses

**Figure 1:**
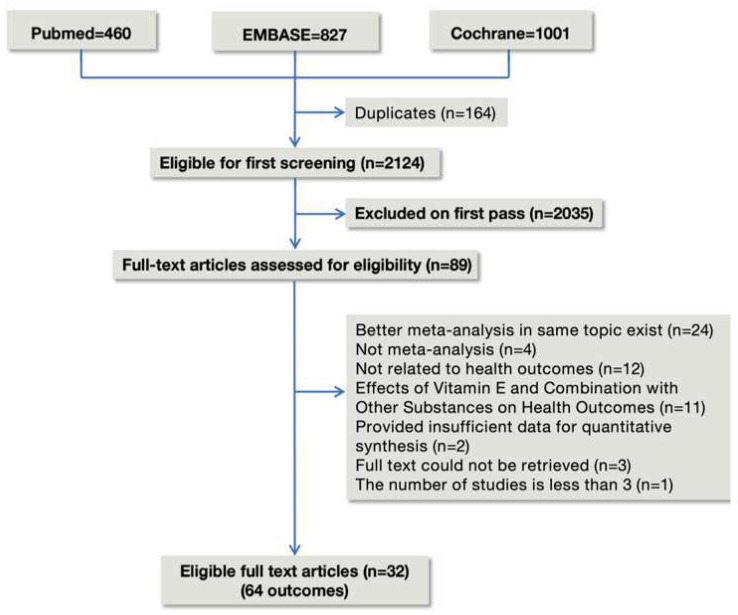
Flowchart of the study selection for the umbrella review.

We carefully reviewed 89 meta-analyses of 2288 articles retrieved from three databases, and we ultimately included 32 publications in our works, as seen in Figure 1. A total of 409 RCTs and 268 observational studies were all from the vitamin E supplementation group. 32 eligible meta-analyses provided 4 types of vitamin E sources and 64 unique health outcomes. Table 1 lists the general characteristics of all included studies. Table S3 provides a list of the 57 excluded articles. We reanalyzed the summary effects of all studies. Finally, detailed summary effects are presented in the form of dichotomous variables and continuous variables. One health outcome (1.6%) was supported by consistent evidence (Class I). 7 results (10.9%) were suggestive evidence (Class III), 23 results (35.9%) had suggestive evidence (Class IV) backing them up, and 33 results (51.6%) were nonsignificant (Class V).

### 3.2. Supplemental Vitamin E Intake

**Figure 2:**
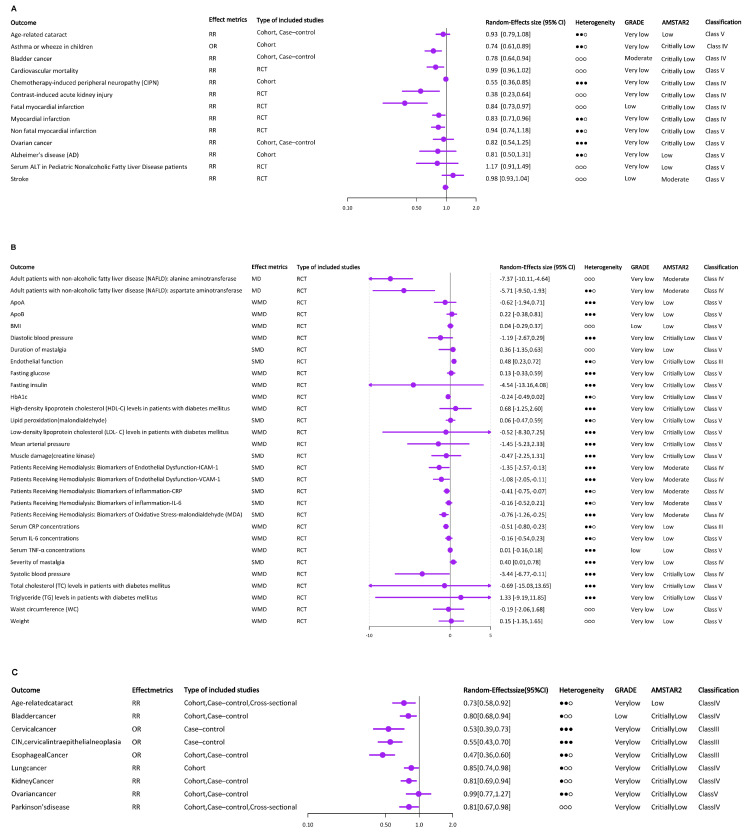

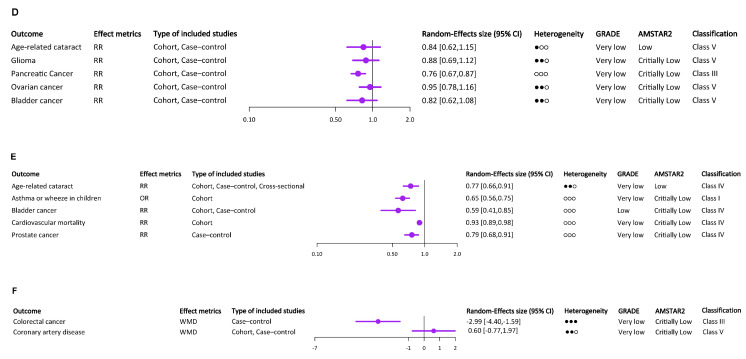
The results and the level of evidence of the effect of vitamin E intake or circulating α-tocopherol and risk of multiple health outcomes. (**A**) The results and the level of evidence of the effect of supplemental vitamin E and risk of health outcomes (binary variable). (**B**) The results and the level of evidence of the effect of supplemental vitamin E and risk of health outcomes (continuous variable). (**C**) The results and the level of evidence of the effect of dietary vitamin E and risk of health outcomes (binary variable). (**D**) The results and the level of evidence of the effect of total vitamin E and risk of health outcomes (binary variable). (**E**) The results and the level of evidence of the effect of circulating α-tocopherol levels and risk of health outcomes (binary variable). (**F**) The results and the level of evidence of the effect of circulating α-tocopherol levels and risk of health outcomes (continuous variable). ○○○ indicates *I*^2^ ≤ 25%, ●○○ indicates 25% < *I*^2^ ≤ 50%, ●●○ indicates 50% < *I*^2^ ≤ 75%, ●●● indicates *I*^2^ ≥ 75%.

Supplemental intake of vitamin E was the most extensively studied area, including 43 health outcomes. We divided these health outcomes into five categories, including cardiovascular system, special population (patients receiving hemodialysis, patients with renal impairment, patients with diabetes mellitus, children, and older adults), cancer, nerve system, and others. A total of 13 of the health outcomes used binary variable, with OR or RR as the effect metrics (Figure 2A). The remaining 30 health outcomes were selected as continuous variables, with mean difference (MD), standardized mean difference (SMD), or weighted mean difference (WMD) as effect metrics (Figure 2B). No studies were rated as consistent evidence (Class I).

#### 3.2.1. Cardiovascular System

Supplemental vitamin E intake reduces mortality from CVD [15]. A meta-analysis of how adult vitamin E consumption affects inflammatory biomarkers provided more evidence for this conclusion. CRP testing is a highly effective cardiovascular mortality predictor. Based on an analysis of 26 RCTs in this meta-analysis, serum CRP concentrations were significantly lower after vitamin E supplementation. In contrast to serum CRP, the effect of vitamin E on IL-6 and TNF-α was not significant [16]. But interestingly, adult ApoA1 and ApoB levels, which are thought to be predictors of CVD, were not significantly affected by vitamin E supplementation [17]. Additionally, a single vitamin E supplement dramatically reduced myocardial infarction when compared to controls. And the decrease in deadly myocardial infarction was what caused this result [18]. Supplemental vitamin E has a protective effect on endothelial function [19]. Among the effects on blood pressure, vitamin E supplements only decreased systolic blood pressure (SBP) [20].

#### 3.2.2. Cancer

Supplemental vitamin E was negatively related with the incidence of bladder cancer [21]. But there was no impact of vitamin E supplementation on the risk of ovarian cancer [22].

#### 3.2.3. Special Population

In patients receiving hemodialysis, supplemental vitamin E may help alleviate oxidative stress and systemic inflammation [23]. Furthermore, vitamin E supplementation combined hydration significantly decreased the incidence of contrast-induced acute kidney damage (CIAKI) [24]. There was no clear relationship between vitamin E supplementation and HbA1c fasting glucose, insulin concentrations [25], and various blood lipid parameters [26] in diabetes mellitus patients. For children, lower probabilities of asthmatic disorders were related to maternal consumption of vitamin E [27]. Supplemental vitamin E, however, was not related to a lower level of blood ALT in children with non-alcoholic fatty liver disease (NAFLD) [28]. One meta-analysis’s results showed supplemental vitamin E consumption had a small effect on the likelihood of developing age-related cataracts (ARC) [29].

#### 3.2.4. Nervous System

It was impossible to determine the precise link between supplementary vitamin E and Alzheimer’s disease (AD) due to follow-up issues with participants, missing data in the original included studies, and the high heterogeneity shown by the forest plots (*I*^2^ = 68%) [30]. Additionally, the effects of supplemental vitamin E on lowering stroke risk were still not supported by statistically meaningful data [31]. Interestingly, the incidence and signs of chemotherapy-induced peripheral neuropathy (CIPN) were improved by vitamin E supplementation [32].

#### 3.2.5. Others

Adult patients with NAFLD who received additional vitamin E had lower liver enzyme levels than those of placebo patients [33]. Additionally, supplemental vitamin E intake can reduce mastalgia’s severity and duration. However, because of the studies’ high degrees of variability, the authors suggest more research utilizing common methodologies based on the CONSORT declaration [34]. Supplementation of vitamin E had no discernible impact on those common indicators of obesity [35].

### 3.3. Dietary Vitamin E Intake

Most of the meta-analyses on the relationship between vitamin E and cancer focused on this section, which contained nice health outcomes, seven of which were cancer related. Interestingly, with the exception of ovarian cancer, which did not have any association with dietary vitamin E, the other included cancers were inversely associated with dietary vitamin E intake [22], including lung cancer [36], kidney cancer [37], cervical cancer, CIN [38], esophageal cancer [39], and bladder cancer [21]. According to dose–response analyses, lung cancer risk decreased by 5% with each 2 mg/d increase in dietary vitamin E consumption. Dietary vitamin E intake was related to ARC and PD in addition to health outcomes related to cancer. It had a strong correlation with a lower incidence of ARC [29]. Etminan et al. discovered that vitamin E consumption in the diet protects against PD [40]. All of the health outcomes used a binary variable, with OR or RR as the effect metrics (Figure 2C). And no studies were rated as consistent evidence (Class I).

### 3.4. Total Vitamin E Intake

Similarly, except for ovarian cancer, all other cancers included were inversely associated with total vitamin E intake, including glioma [41], pancreatic cancer [42], and bladder cancer [21]. A lower incidence of both cancer and ARC was strongly associated with total vitamin E consumption [29]. All of the health outcomes used a binary variable, with RR as the effect metrics (Figure 2D). And only studies on the link between pancreatic cancer and total vitamin E intake were rated as suggestive evidence (Class III).

### 3.5. Circulating α-Tocopherol Levels

Circulating α-tocopherol’s results are shown in Figure 2E,F. Seven meta-analyses examined the association between circulating α-tocopherol levels and health outcomes, including ARC [29], asthma or wheeze in children [27], bladder cancer [21], cardiovascular mortality [15], colorectal cancer [43], CVD [44], and prostate cancer [45]. Except that circulating α-tocopherol levels did not significantly reduce the risk of CVD, there was a protective association between circulating α-tocopherol levels and the remaining six health outcomes. The association between circulating α-tocopherol levels and asthma or wheeze in children was rated as consistent evidence (Class I).

### 3.6. Heterogeneity between Primary Studies

We reanalyzed *I*^2^ values, which were used to assess study heterogeneity, for the included meta-analyses by random effects model (Table 1). We reanalyzed 22 health outcomes from the included meta-analyses and found significant heterogeneity (*I*^2^ > 75%); furthermore, 21 health outcomes from included meta-analyses detected moderate heterogeneity (*I*^2^ > 50%). A low level of heterogeneity (*I*^2^ > 25%) was observed in four health outcomes from included meta-analyses. The remaining 17 health outcomes from included meta-analyses showed no heterogeneity (*I*^2^ > 0%).

### 3.7. Publication Bias of Included Studies

By using Egger’s test, we identified publication bias in 32 health outcomes. There was statistical evidence of publication bias for four health outcomes (Table 1). This included bladder cancer (dietary vitamin E), esophageal cancer (dietary vitamin E), pancreatic cancer (total vitamin E intake), and asthma or wheeze in children (circulating α-tocopherol levels).

### 3.8. AMSTAR and GRADE Classification of Included Studies

Using online AMSTAR 2, we evaluated the methodological quality. In Table S4, for each of the included meta-analyses, unique AMSTAR data were shown. And they were rated as having high, moderate, poor, and critically low quality. With regard to quality of evidence, a total of three meta-analyses (9.4%) were classified as moderate. Seven meta-analyses (21.9%) were graded as low quality. The remaining 22 meta-analyses (68.8%) were classified as critically low. Due to the small sample size, bias risk, inconsistent results, and imprecision, none of them were given a high ranking. Detailed GRADE scores for every health outcome were summarized in Table S5.

## 4. Discussion

This detailed comprehensive review included 32 publications including 4 types of vitamin E source and 64 unique health outcomes from 409 RCTs and 268 observational studies. The role of vitamin E has been studied in a wide range of health outcomes, including cancers, CVD, autoimmune diseases, renal diseases, metabolic diseases, respiratory diseases, and aging diseases. Among them, the most studied diseases are still focused on cancers and CVD. For the meta-analysis of RCTs, outcomes were only limited to supplemental vitamin E. Only six meta-analyses of health outcomes in supplemental vitamin E group came from observational studies, including ARC, AD, cardiovascular mortality, ovarian cancer, bladder cancer, and childhood asthmatic diseases; among them, the association between the first four health outcomes and supplemental vitamin E was not significant, and the last two health outcomes had beneficial associations with supplemental vitamin E. The remaining three meta-analyses of vitamin E sources and related health outcomes were based on original studies from observational studies.

Most meta-analyses that have included only observational studies have shown associations that are limited by the false positive caveats that accompany the evidence from observational studies, and very few, if any, may translate to effective interventions when tested in RCTs. In addition, not all meta-analyses of included RCTs can yield accurate associations, especially when the sample size of the RCTs is limited and the level of statistical significance is weak. For example, although the umbrella review suggests that supplemental vitamin E may benefit indicators of oxidative stress and inflammation in hemodialysis patients, given the limited quality of the included RCTs and the heterogeneity of the results reported in the available trials, we still favor following the new guidelines for nutrition in CKD [46], which support that there is insufficient proof to advise them to take vitamin E supplements. According to the results of this umbrella review, there was only consistent evidence of a beneficial association between circulating α-tocopherol levels and asthma or wheeze in children in both randomized and observational evidence. However, given that this meta-analysis included observational studies, both the Grade and AMSTAR2 grades were very low.

Among all the health outcomes associated with vitamin E intake or circulating α-tocopherol, this umbrella review demonstrated that vitamin E demonstrated a protective effect in 31 health outcomes, including 16 related to supplemental vitamin E (including alanine aminotransferase and spartate aminotransferase indicators in adult patients with NAFLD, oxidative stress and inflammation indicators in hemodialysis patients, childhood asthma, bladder cancer, CIPN, contrast-induced acute kidney injury, endothelial function, systolic blood pressure, myocardial infarction and fatal myocardial infarction, and severity of mastalgia), 8 related to dietary vitamin E (including ARC, Parkinson’s disease, and various cancers: bladder cancer, cervical cancer, CIN, cervical intraepithelial neoplasia, esophageal cancer, lung cancer, and kidney cancer), 1 related to total vitamin E (pancreatic cancer), and 6 related to circulating α-tocopherol levels (including ARC, asthma or wheeze in children, cardiovascular mortality, and 3 types of cancers: bladder cancer, colorectal cancer, and prostate cancer). Vitamin E did not show a significant effect on the remaining 33 health outcomes. There were 27, 1, 4, and 1 health outcomes associated with supplemental vitamin E, dietary vitamin E, total vitamin E, and circulating α-tocopherol levels, respectively. No negative associations were found between vitamin E intake or circulating α-tocopherol and health outcomes in this umbrella review. This result also broadens the use of vitamin E for some specific health outcomes. For example, this umbrella review suggested that supplemental vitamin E was not related with reduced level of serum ALT in pediatric NAFLD patients. However, the 2018 guidelines suggested appropriate doses of vitamin E for children with biopsied NAFLD, although further study is needed before vitamin E can be widely recommended in clinical practice [47]. This also indicates that the use of appropriate doses of vitamin E in pediatric NAFLD patients is acceptable until more robust research evidence is available.

Additionally, more than half of the meta-analyses of observational studies (17/27) reported nominally statistically significant protective associations. However, meta-analyses of RCTs reported nominally statistically significant pooled results for only 15 of 37 healthy outcomes (including alanine aminotransferase and spartate aminotransferase indicators in adult patients with NAFLD, oxidative stress and inflammation indicators in hemodialysis patients, serum CRP concentrations, CIPN, contrast-induced acute kidney injury, endothelial function, systolic blood pressure, myocardial infarction and fatal myocardial infarction, and severity of mastalgia). This result may be due to the lower statistical power of the meta-analysis of included RCTs and the fact that the results of RCTs are more conservative than those of observational studies. In the results of this umbrella review, none of the very promising results found in meta-analyses of observational studies have been tested in meta-analyses of RCTs.

Cancer and CVD are the two most studied diseases in this umbrella review, and the underlying mechanism of vitamin E’s role in these two diseases may be closely related to its powerful anti-inflammatory and antioxidant abilities [48]. According to the 2014 USPSTF, it is insufficient to support the claim that supplementing with vitamin E does not lower the development either of cardiovascular disease or cancer in healthy populations without known nutritional deficiencies. Adequate evidence shows that vitamin E supplementation has either little or no substantial harm. Therefore, the USPSTF draws the conclusion that supplementing with vitamin E has no discernible advantage in terms of preventing cancer or cardiovascular disease [49]. The revised USPSTF recommendation statement from 2022 reiterates the main finding of the statement from 2014, but it further urges intake of foods high in antioxidant vitamins and other nutrients for general health and wellness as well as for particular preventative purposes [50]. In this umbrella review, although no studies were classified as consistent or highly suggestive evidence, dietary vitamin E showed positive protective effects on most cancer types (except for ovarian cancer). Therefore, the conclusions of this umbrella review are also consistent with USPSTF recommendations. With the deepening of related research, a growing body of preclinical research shows that the structure of vitamin E is a crucial element in the prevention of cancer caused by vitamin E. Mechanistic studies, in particular, have demonstrated that γ, δ, γ, and δ-tocotrienols are significantly more potent than α-tocopherol in blocking many cancer-prevention pathways [51]. In contrast to α-tocopherol, which is largely unmetabolized, γ-tocopherol, δ-tocopherol, γ-tocotrienol, and δ-tocotrienol are easily metabolized, and their long-chain metabolites 13′-COOHs are special dual inhibitors of COXs and 5-LOX and have stronger anti-inflammatory and anticancer effects than some vitamers that are not metabolized [52,53]. Most dietary vitamin E or vitamin E supplements exert their effects in the human body in the form of α-tocopherol [54]. Therefore, this may partially explain why the risk of ovarian cancer was not associated with supplemental or dietary vitamin E in this umbrella review. This also suggests that future RCTs should focus more on the effect of intake of one specific vitamin E isoforms on cancer.

This umbrella review suggests that supplemental vitamin E intake improves endothelial function, reduces myocardial infarction, and reduces mortality from CVD. In terms of cardiovascular mortality, the circulating α-tocopherol levels suggest the same conclusion. However, circulating α-tocopherol levels did not appear to be related to the risk of CVD. This paradoxical and interesting phenomenon caught our attention. According to the existing epidemiological studies, increasing vitamin E intake can reduce the risk of CVD. However, this effect was only shown in people who used high-dose vitamin E supplements daily for more than 2 years [55]. It could be concluded that the dose of vitamin E intake was crucial in the process of its effect. This might be related to the levels of circulating α-tocopherol in food or supplements, which were eventually mainly utilized by the human body to exert their effects. It might also be that high doses of vitamin E intake ensure the enrichment of other isoforms of tocopherols or tocotrienols in human circulation to exert a protective effect against CVD. For example, some studies confirmed an inverse association between γ-tocopherol supplementation alone and the risk of coronary heart disease [56]. Therefore, future studies should fully consider the possible effect of vitamin E supplementation dose and effective tocopherols or tocotrienols circulating concentrations on outcomes when developing individualized medication strategies for CVD patients.

This umbrella review has systematically integrated the current evidence about the associations between vitamin E intakes or circulating α-tocopherol and multiple health outcomes for the first time. Generally, effect metrics with 95% CI are used to determine the link between exposure and outcomes, but if studies demonstrate significant heterogeneity or publication bias, this connection has to be questioned [57]. We were the first to evaluate the quality and strength of the evidence from all included meta-analyses using the AMSTAR2 and GRADE classification methods. Nevertheless, some possible limitations should be noted. Firstly, a relatively large number of the meta-analyses were “Critically Low” in the AMSTAR2 classification as well as “Very low” in GRADE categorizations. This phenomenon was largely caused by many studies not assessing the potential impact of risk of bias in individual studies on meta-analysis results and did not consider the risk of bias in individual studies when discussing review results. For GARDE categorization, serious imprecision derived from limited studies numbers and population numbers as well as considerate confidence intervals for the estimates of effect sizes. Undetected publication bias is due to the fact that most of the original studies did not report funnel plots or Egger *p* values. Furthermore, few studies met the upgrading items, including a relatively large magnitude of effect and beneficial plausible confounding factors. There is no statistical association between AMSTAR2 and GRADE, since the rating domains differ. Secondly, small populations and studies were included in some of the eligible meta-analyses, which likely contributed to publication bias. Another limitation of our study is that we were not able to accurately assess vitamin E intake from different sources across the studies because we were not able to extract data on vitamin E intake. In addition, the intake of the sources of vitamin E intake varied among the various original studies included in the meta-analysis. For example, the umbrella review showed that adding extra vitamin E to the diet of adults with NAFLD reduced their liver enzyme levels. Given the current lack of approved drugs to treat NAFLD, the mainstay of patient care for NAFLD remains tailoring diet and other lifestyle changes to the needs of each individual patient. Vitamin E is often considered as the first line of defense against NAFLD when dietary and other lifestyle changes are not enough [58]. However, with the deepening of research, some studies support that the long-term use of vitamin E in NAFLD patients may affect the mortality of patients [59]. It can be concluded that the choice of safe therapeutic dose of vitamin E still needs to be further explored. The specific dose of vitamin E supplementation should also be considered in the design of future studies. In this umbrella review, most meta-analyses produced pooled effects of the original studies, which measured vitamin E exposure by whether vitamin E was consumed or not. This may also partly explain the source of heterogeneity in the meta-analysis. Nevertheless, this umbrella review provides a bird’s eye view of the available evidence examining the association between vitamin E intakes or circulating α-tocopherol and multiple health outcomes and a comprehensive assessment of the strength of the available evidence and can indicate potential priorities for future research. In the future, there is still a need for clinicians and trialists to conduct RCTs to fill the current gap of high-quality evidence between vitamin E and most health outcomes.

In conclusion, this umbrella review extensively studied a wide range of relationships between vitamin E intake or circulating α-tocopherol and various health outcomes. There are some indications that vitamin E may be associated with several health outcomes. However, no firm general conclusion can be drawn about benefits. Associations between dietary vitamin E, total vitamin E and circulating α-tocopherol levels, and relevant health outcomes have only been validated in observational studies, but these associations were either not statistically significant or not verified in meta-analyses that included RCTs. RCTs for cancer-related outcomes are clearly lacking. Additionally, evidence from the meta-analysis of RCTs suggested that supplemental vitamin E was associated with 15 health outcomes. These health outcomes may not need to be extensively investigated in future studies. However, for health outcomes with low quality of evidence, the association with vitamin E still needs further research and better designed trials to draw firm conclusions.

## Figures and Tables

**Table 1:** The results and the level of evidence of the effect of vitamin E and risk of health outcomes.

Outcome	Author	Year	Effect Metrics	Number of Included Studies	Type of Included Studies	Egger *p* Value	Random-Effects Size (95% CI)	*p* Value	I2(%)	Q Test *p* Value	Tau2	95% Prediction Interval	GRADE	AMSTAR2	Classification
**Supplemental vitamin E**															
**Adult patients with non-alcoholic fatty liver disease (NAFLD): alanine aminotransferase**	Vadarlis	2021	MD	7	RCT	NA	−7.37 [−10.11,−4.64]	1.29495 × 10^−7^	0.00	0.4493543	0	−10.96 to −3.78	Very low	Moderate	Class IV
**Adult patients with non-alcoholic fatty liver disease (NAFLD): aspartate aminotransferase**	Vadarlis	2021	MD	7	RCT	NA	−5.71 [−9.50,−1.93]	0.0031	67.99	0.004613523	14.29	−16.62 to 5.20	Very low	Moderate	Class IV
**Age-related cataract**	Zhang	2015	RR	10	Cohort, Case–control	NA	0.93 [0.79,1.08]	0.34	73.27	0.000101994	0.033	0.59 to 1.47	Very low	Low	Class V
**Alzheimer’s disease (AD)**	Wang	2021	RR	5	Cohort	0.66	0.81 [0.50,1.31]	0.39	68.06	0.01386352	0.18	0.17 to 3.84	Very low	Low	Class V
**ApoA**	Hamedi	2021	WMD	4	RCT	0.73	−0.62 [−1.94,0.71]	0.36	93.53	4.75651 × 10^−10^	1.68	−6.90 to 5.66	Very low	Low	Class V
**ApoB**	Hamedi	2021	WMD	7	RCT	0.55	0.22 [−0.38,0.81]	0.48	84.83	5.58564 × 10^−7^	0.51	−1.78 to 2.21	Very low	Low	Class V
**Asthmatic diseases in children**	Wu	2018	OR	10	Cohort	0.13	0.74 [0.61,0.89]	0.0013	73.28	0.000101632	0.042	0.44 to 1.24	Very low	Critially Low	Class IV
**Bladder cancer**	Chen	2015	RR	7	Cohort, Case–control	0.39	0.78 [0.64,0.94]	0.01	0.00	0.4242131	0	0.60 to 1.00	Moderate	Critially Low	Class IV
**BMI**	Emami	2021	WMD	27	RCT	0.38	0.04 [−0.29,0.37]	0.82	0.00	0.9994954	0	−0.30 to 0.38	Low	Low	Class V
**Cardiovascular mortality**	Jayedi	2019	RR	7	Cohort	NA	0.99 [0.96,1.02]	0.54	0.00	0.6680903	0	0.95 to 1.03	Very low	Critially Low	Class V
**Chemotherapy-induced peripheral neuropathy (CIPN)**	Chen	2021	RR	8	RCT	NA	0.55 [0.36,0.85]	0.0069	77.14	7.29272 × 10^−5^	0.23	0.15 to 2.01	Very low	Critially Low	Class IV
**Contrast-induced acute kidney injury**	Cho	2017	RR	3	RCT	NA	0.38 [0.23,0.64]	0.0003	0.00	0.6189431	0	0.01 to 11.37	Very low	Critially Low	Class IV
**Diastolic blood pressure**	Emami	2019	WMD	17	RCT	NA	−1.19 [−2.67,0.29]	0.11	89.79	3.59574 × 10^−25^	5.95	−6.64 to 4.25	Very low	Critially Low	Class V
**Duration of mastalgia**	Hajizadeh	2019	SMD	3	RCT	NA	0.36 [−1.35,0.63]	0.47	0.00	0.8534552	0	−6.78 to 6.06	Very low	Low	Class V
**Endothelial function**	Siervo	2015	SMD	27	RCT	0.61	0.48 [0.23,0.72]	0.00012	64.48	2.23892 × 10^−6^	0.26	−0.60 to 1.55	Very low	Critially Low	Class III
**Fasting glucose**	Xu	2014	WMD	12	RCT	0.87	0.13 [−0.33,0.59]	0.57	88.05	6.56948 × 10^−15^	0.36	−1.31 to 1.57	Very low	Critially Low	Class V
**Fasting insulin**	Xu	2014	WMD	6	RCT	0.27	−4.54 [−13.16,4.08]	0.3	88.55	2.71116 × 10^−8^	59.27	−29.15 to 20.08	Very low	Critially Low	Class V
**Fatal myocardial infarction**	Loffredo	2015	RR	6	RCT	0.09	0.84 [0.73,0.97]	0.015	0.00	0.5299839	0	0.69 to 1.02	Low	Critially Low	Class IV
**HbA1c**	Xu	2014	WMD	12	RCT	0.46	−0.24 [−0.49,0.02]	0.066	66.89	0.000484398	0.11	−1.02 to 0.55	Very low	Critially Low	Class V
**High-density lipoprotein cholesterol (HDL-C) levels in patients with diabetes mellitus**	Mohammad	2021	WMD	11	RCT	NA	0.68 [−1.25,2.60]	0.49	85.37	9.19549 × 10^−11^	7.14	−5.76 to 7.12	Very low	Critially Low	Class V
**Lipid peroxidation(malondialdehyde)**	Stepanyan	2014	SMD	10	RCT	NA	0.06 [−0.47,0.59]	0.83	56.75	0.01351643	0.41	−1.55 to 1.66	Very low	Critially Low	Class V
**Low-density lipoprotein cholesterol (LDL- C) levels in patients with diabetes mellitus**	Mohammad	2021	WMD	11	RCT	NA	−0.52 [−8.30,7.25]	0.89	89.00	3.60121 × 10^−15^	139.75	−28.73 to 27.68	Very low	Critially Low	Class V
**Mean arterial pressure**	Emami	2019	WMD	7	RCT	NA	−1.45 [−5.23,2.33]	0.45	79.02	7.2278 × 10^−5^	13.32	−12.07 to 9.16	Very low	Critially Low	Class V
**Muscle damage(creatine kinase)**	Stepanyan	2014	SMD	8	RCT	NA	−0.47 [−2.25,1.31]	0.61	91.31	1.08004 × 10^−14^	5.87	−6.80 to 5.87	Very low	Critially Low	Class V
**Myocardial infarction**	Loffredo	2015	RR	8	RCT	0.09	0.83 [0.71,0.96]	0.014	58.67	0.01782035	0.022	0.55 to 1.25	Very low	Critially Low	Class IV
**Non fatal myocardial infarction**	Loffredo	2015	RR	7	RCT	0.15	0.94 [0.74,1.18]	0.57	63.97	0.01064533	0.047	0.50 to 1.77	Very low	Critially Low	Class V
**Ovarian cancer**	Leng	2019	RR	5	Cohort, Case–control	NA	0.82 [0.54,1.25]	0.35	82.33	0.000149404	0.19	0.18 to 3.78	Very low	Critially Low	Class V
**Patients Receiving Hemodialysis: Biomarkers of Endothelial Dysfunction-ICAM-1**	Nguyen	2021	SMD	4	RCT	0.37	−1.35 [−2.57,−0.13]	0.03	89.01	5.12701 × 10^−6^	1.28	−6.91 to 4.21	Very low	Moderate	Class IV
**Patients Receiving Hemodialysis: Biomarkers of Endothelial Dysfunction-VCAM-1**	Nguyen	2021	SMD	3	RCT	0.83	−1.08 [−2.05,−0.11]	0.029	80.38	0.006122837	0.58	−12.60 to 10.44	Very low	Moderate	Class IV
**Patients Receiving Hemodialysis: Biomarkers of inflammation-CRP**	Nguyen	2021	SMD	9	RCT	0.58	−0.41 [−0.75,−0.07]	0.017	63.71	0.0048335	0.16	−1.45 to 0.63	Very low	Moderate	Class IV
**Patients Receiving Hemodialysis: Biomarkers of inflammation-IL-6**	Nguyen	2021	SMD	5	RCT	0.23	−0.16 [−0.52,0.21]	0.4	52.96	0.07476872	0.089	−1.27 to 0.96	Very low	Moderate	Class V
**Patients Receiving Hemodialysis: Biomarkers of Oxidative Stress-malondialdehyde (MDA)**	Nguyen	2021	SMD	6	RCT	0.059	−0.76 [−1.26,−0.25]	0.0032	76.58	0.000694967	0.3	−2.43 to 0.92	Very low	Moderate	Class IV
**Serum ALT in Pediatric Nonalcoholic Fatty Liver Disease patients**	Sarkhy	2014	RR	4	RCT	NA	1.17 [0.91,1.49]	0.22	0.00	0.7688677	0	0.68 to 1.99	Very low	Low	Class V
**Serum CRP concentrations**	Asbaghi	2020	WMD	36	RCT	NA	−0.51 [−0.80,−0.23]	0.00042	59.82	2.48248 × 10^−6^	0.23	−1.53 to 0.50	Very low	Low	Class III
**Serum IL-6 concentrations**	Asbaghi	2020	WMD	21	RCT	NA	−0.16 [−0.54,0.23]	0.42	74.35	8.60757 × 10^−9^	0.4	−1.54 to 1.23	Very low	Low	Class V
**Serum TNF-α concentrations**	Asbaghi	2020	WMD	19	RCT	NA	0.01 [−0.16,0.18]	0.93	78.90	9.87231 × 10^−11^	0.062	−0.55 to 0.56	low	Low	Class V
**Severity of mastalgia**	Hajizadeh	2019	SMD	7	RCT	NA	0.40 [0.01,0.78]	0.042	79.06	7.06353 × 10^−5^	0.2	−0.86 to 1.66	Very low	Low	Class IV
**Stroke**	Loh	2020	RR	12	RCT	0.25	0.98 [0.93,1.04]	0.59	0.00	0.4715554	0	0.92 to 1.05	Low	Moderate	Class V
**Systolic blood pressure**	Emami	2019	WMD	23	RCT	NA	−3.44 [−6.77,−0.11]	0.043	94.00	2.93369 × 10^−64^	55.23	−19.29 to 12.42	Very low	Critially Low	Class IV
**Total cholesterol (TC) levels in patients with diabetes mellitus**	Mohammad	2021	WMD	12	RCT	NA	−0.69 [−15.03,13.65]	0.92	96.32	1.31883 × 10^−57^	597.03	−57.52 to 56.14	Very low	Critially Low	Class V
**Triglyceride (TG) levels in patients with diabetes mellitus**	Mohammad	2021	WMD	12	RCT	NA	1.33 [−9.19,11.85]	0.8	76.73	1.92346 × 10^−6^	186.61	−31.37 to 34.03	Very low	Critially Low	Class V
**Waist circumference (WC)**	Emami	2021	WMD	7	RCT	0.072	−0.19 [−2.06,1.68]	0.84	0.00	0.9777026	0	−2.64 to 2.26	Very low	Low	Class V
**Weight**	Emami	2021	WMD	16	RCT	0.63	0.15 [−1.35,1.65]	0.845	0.00	0.9998251	0	−1.49 to 1.79	Very low	Low	Class V
**Dietary vitamin E**															
**Age-related cataract**	Zhang	2015	RR	8	Cohort, Case–control, Cross-sectional	NA	0.73 [0.58,0.92]	0.0073	69.13	0.001942455	0.066	0.37 to 1.46	Very low	Low	Class IV
**Bladder cancer**	Chen	2015	RR	9	Cohort, Case–control	0.01	0.80 [0.68,0.94]	0.0077	32.97	0.1541656	0.02	0.54 to 1.18	Low	Critially Low	Class IV
**Cervical cancer**	Hu	2017	OR	9	Case–control	NA	0.53 [0.39,0.73]	7.32494 × 10^−5^	77.64	1.92167 × 10^−5^	0.17	0.19 to 1.50	Very low	Critially Low	Class III
**CIN, cervical intraepithelial neoplasia**	Hu	2017	OR	17	Case–control	NA	0.55 [0.43,0.70]	1.2434 × 10^−6^	78.10	2.88363 × 10^−9^	0.2	0.21 to 1.46	Very low	Critially Low	Class III
**Esophageal Cancer**	Cui	2018	OR	14	Cohort, Case–control	0.008	0.47 [0.36, 0.60]	3.77685 × 10^−9^	66.95	0.000177109	0.13	0.20 to 1.09	Very low	Critially Low	Class III
**Lung cancer**	Zhu	2017	RR	11	Cohort	0.25	0.85 [0.74, 0.98]	0.03	41.83	0.07023408	0.023	0.58 to 1.25	Very low	Critially Low	Class IV
**Kidney Cancer**	Shen	2015	RR	13	Cohort, Case–control	0.93	0.81 [0.69,0.94]	0.0065	49.22	0.02282328	0.035	0.52 to 1.26	Very low	Critially Low	Class IV
**Ovarian cancer**	Leng	2019	RR	8	Cohort, Case–control	NA	0.99 [0.77,1.27]	0.95	52.76	0.0383885	0.066	0.49 to 2.00	Very low	Critially Low	Class V
**Parkinson’s disease**	Etminan	2005	RR	7	Cohort, Case–control, Cross-sectional	NA	0.81 [0.67,0.98]	0.028	0.00	0.6156204	0	0.63 to 1.04	Very low	Critially Low	Class IV
**Total vitamin E**															
**Age-related cataract**	Zhang	2015	RR	4	Cohort, Case–control	NA	0.84 [0.62,1.15]	0.29	47.08	0.1288757	0.047	0.26 to 2.69	Very low	Low	Class V
**Glioma**	Qin	2014	RR	12	Cohort, Case–control	0.51	0.88 [0.69,1.12]	0.31	64.87	0.000983177	0.11	0.40 to 1.94	Very low	Critially Low	Class V
**Pancreatic Cancer**	Peng	2015	RR	12	Cohort, Case–control	0.049	0.76 [0.67,0.87]	0.000053	19.80	0.2548871	0.0091	0.59 to 0.99	Very low	Critially Low	Class III
**Ovarian cancer**	Leng	2019	RR	11	Cohort, Case–control	NA	0.95 [0.78,1.16]	0.64	53.19	0.01869907	0.057	0.53 to 1.72	Very low	Critially Low	Class V
**Bladder cancer**	Chen	2015	RR	6	Cohort, Case–control	0.35	0.82 [0.62,1.08]	0.15	55.17	0.04842951	0.063	0.37 to 1.82	Very low	Critially Low	Class V
**Circulating α-tocopherol levels**															
**Age-related cataract**	Zhang	2015	RR	17	Cohort, Case–control, Cross-sectional	NA	0.77 [0.66,0.91]	0.0019	52.09	0.006544955	0.043	0.48 to 1.24	Very low	Low	Class IV
**Asthma or wheeze in children**	Wu	2018	OR	7	Cohort	0.035	0.65 [0.56,0.75]	1.37859 × 10^−8^	0.00	0.4474548	0	0.53 to 0.79	Very low	Critially Low	Class I
**Bladder cancer**	Chen	2015	RR	4	Cohort, Case–control	0.39	0.59 [0.41,0.85]	0.0046	0.00	0.607105	0	0.26 to 1.32	Low	Critially Low	Class IV
**Cardiovascular mortality**	Jayedi	2019	RR	4	Cohort	NA	0.93 [0.89,0.98]	0.0043	9.15	0.3473236	0.00055	0.81 to 1.08	Very low	Critially Low	Class IV
**Colorectal cancer**	Dong	2017	WMD	11	Case–control	NA	−2.99 [−4.40,−1.59]	2.94537 × 10^−5^	94.98	2.50467 × 10^−37^	3.01	−7.24 to 1.25	Very low	Critially Low	Class III
**CVD**	Li	2016	WMD	12	Cohort, Case–control	0.54	0.60 [−0.77,1.97]	0.39	64.44	0.001129312	3.36	−3.77 to 4.97	Very low	Critially Low	Class V
**Prostate cancer**	Cui	2014	RR	9	Case–control	0.08	0.79 [0.68,0.91]	0.0015	12.45	0.3308022	0.0062	0.61 to 1.02	Very low	Critially Low	Class IV

## Data Availability

No new data were created or analyzed in this study. Data sharing is not applicable to this article.

## Supplementary Materials

The following supporting information can be downloaded at: https://www.mdpi.com/article/10.3390/nu15153301/s1, Table S1. Search strategy; Table S2. Summary of evidence classification for meta-analyses; Table S3. The list of excluded meta-analyses by full text screening with exclusion reason; Table S4. Assessments of AMSTAR scores; Table S5. GRADE classification of quality of evidence of all included meta-analyses.
