# Supplementary material for: Vitamin E and Multiple Health Outcomes: An Umbrella Review of Meta-Analyses
Source: Nutrients. 2023 Jul 25;15(15):3301. doi: 10.3390/nu15153301 (PMC10421296; doi:10.3390/nu15153301)
Supplement: Supplementary file 1 [file nutrients-15-03301-s001.zip › Table S1.pdf]

Supplementary Table S1. Search strategy.

Medline (through PubMed)

Query #	Query	Result
1.	Tocopherols[Mesh] OR Tocopherol[tiab] OR Tocovital[tiab] OR Uno-Vit[tiab] OR Uno Vit[tiab] OR UnoVit[tiab] OR Vita-Plus E[tiab] OR Vita Plus E[tiab] OR VitaPlus E[tiab] OR Vitagutt Vitamin E[tiab] OR Vitamin E, Vitagutt[tiab] OR Vitamin E AL[tiab] OR Vitamin E Natur[tiab] OR Vitamin E Sanum[tiab] OR Vitamin E Suspension[tiab] OR Vitamin-E Dragees[tiab] OR Vitamin E Dragees[tiab] OR Vitamin-E EVI-MIRALE[tiab] OR Vitamin E EVI MIRALE[tiab] OR VitaminE EVIMIRALE[tiab] OR Vitamine E GNR[tiab] OR Vitazell[tiab] OR Detulin[tiab] OR E Vitamin E[tiab] OR Vitamin E-mp[tiab] OR Vitamin E mp[tiab] OR Vitamin Emp[tiab] OR Vitamin E[tiab] OR E-ferol[tiab] OR E-Mulsin[tiab] OR E Mulsin[tiab] OR E-Vicotrat[tiab] OR E Vicotrat[tiab] OR E-Vitamin-Ratiopharm[tiab] OR Ecoro[tiab] OR Elex Verla[tiab] OR Embial[tiab] OR Evion[tiab] OR Ephynal[tiab] OR Eplonat[tiab] OR Equivit E[tiab] OR EUNOVA Vitamin E[tiab] OR Antioxidans E-Hevert[tiab] OR Eusovit[tiab] OR Hydrovit E[tiab] OR Micorvit E[tiab] OR Malton E[tiab] OR Mowivit Vitamin E[tiab] OR Vitamin E, Mowivit[tiab] OR Puncto E[tiab] OR Richtavit E[tiab] OR Sanavitan S[tiab] OR Snow-E Muscle, Energy and Feritility[tiab] OR Dal-E[tiab] OR Dal E[tiab] OR Abortosan[tiab] OR Aquasol E[tiab] OR Auxina E[tiab] OR Bio E[tiab] OR Biopto-E[tiab] OR Biosan[tiab] OR Lasar[tiab] OR Bioweyxin[tiab] OR Davitamon[tiab] OR Dermorelle[tiab] OR Spondyvit[tiab] OR Tocolion[tiab] OR Tocopa[tiab] OR Tocopharm[tiab] OR Vibolex[tiab] OR Vit E hydrosol[tiab] OR Vit. E Stada[tiab] OR Vita-E[tiab] OR Vita E[tiab] OR VitaE[tiab] OR Tocopherol Bayer[tiab] OR Togasan Vitamin E[tiab] OR Vitamin E, Togasan[tiab] OR Unique E[tiab] OR Antioxidants*[tiab]	677,682
2.	meta-analy*[all fields] OR meta-analysis[publication type] OR "Meta-Analysis as Topic"[Mesh]	264,678
3.	1 AND 2	2,097

Embase (Ovid platform)

Query #	Query	Result
1.	(Tocopherols OR Tocopherol OR Tocovital OR Uno-Vit OR Uno Vit OR UnoVit OR Vita-Plus E OR Vita Plus E OR VitaPlus E OR Vitagutt Vitamin E OR Vitamin E, Vitagutt OR Vitamin E AL OR Vitamin E Natur OR Vitamin E Sanum OR Vitamin E Suspension OR Vitamin-E Dragees OR Vitamin E Dragees OR Vitamin-E EVI-MIRALE OR Vitamin E EVI MIRALE OR VitaminE EVIMIRALE OR Vitamine E GNR OR Vitazell OR Detulin OR E Vitamin E OR Vitamin E-mp OR Vitamin E mp OR Vitamin Emp OR Vitamin E OR E-ferol OR E-Mulsin OR E Mulsin OR E-Vicotrat OR E Vicotrat OR E-Vitamin-Ratiopharm OR Ecoro OR Elex Verla\ OR Embial OR Evion OR Ephynal OR Eplonat OR Equivit E OR EUNOVA Vitamin E OR Antioxidans E-Hevert OR Eusovit OR Hydrovit E OR Micorvit E OR Malton E OR Mowivit Vitamin E OR Vitamin E, Mowivit OR Puncto E OR Richtavit E OR Sanavitan S OR Snow-E Muscle, Energy and Feritility OR Dal-E OR Dal E OR Abortosan OR Aquasol E OR Auxina E OR Bio E OR Biopto-E OR Biosan OR Lasar OR Bioweyxin OR Davitamon OR Dermorelle OR Spondyvit OR Tocolion OR Tocopa OR Tocopharm OR Vibolex OR Vit E hydrosol OR Vit. E Stada OR Vita-E OR Vita E OR VitaE OR Tocopherol Bayer OR Togasan Vitamin E OR Vitamin E, Togasan OR Unique E OR Antioxidants OR alpha-Tocopherol OR beta-Tocopherol OR gamma-Tocopherol).mp	27,421
2.	meta-analyses.mp	350,547
3.	1 AND 2	1,095

Cochrane Database of Systematic Reviews (Ovid platform)

Query #	Query	Result
1.	MeSH descriptor: [Tocopherols] explode all trees	769
2.	(Aquasol E or Vitamin E Suspension or Malton E or Equivit E or Vitamin E Natur or Spondyvit or Togasan Vitamin E or Vitamin E, Togasan or Mowivit Vitamin E or Vitamin E, Mowivit or Vit. E Stada or Unique E or Tocopherol or Ecoro or Eplonat or Snow-E Muscle, Energy & Feritility or E-Vicotrat or E Vicotrat or Vitagutt Vitamin E or Vitamin E, Vitagutt or Tocovital or Antioxidans E-Hevert or EUNOVA Vitamin E or Bioweyxin or Uno-Vit or UnoVit or Uno Vit or Eusovit or E Mulsin or E-Mulsin or Biopto-E or Puncto E or Lasar or Biosan or Vitamin E Sanum or Elex Verla or Detulin or Vitamin-E Dragees or Vitamin E Dragees or Vibolex or Ephynal or Micorvit	11951

	E or Hydrovit E or Dal E or Dal-E or Bio E or Vitamin E-mp or Vitamin E mp or E Vitamin E or Vitamin Emp or Tocopa or Vitamin-E EVI-MIRALE or Vitamin E EVI MIRALE or VitaminE EVIMIRALE or Sanavitan S or Tocopherol Bayer or Abortosan or Davitamon or Vitamin E AL or Dermorelle or Evion or Embial or Auxina E or Vitamine E GNR or Vit E hydrosol or E-Vitamin-Ratiopharm or Richtavit E or VitaPlus E or Vita-Plus E or Vita Plus E or Vitazell):ti,ab,kw	
3.	1 OR 2	12720
4.	(meta-analyses):ti,ab,kw	19987
5.	3 AND 4	569
