# Supplementary material for: Vitamin E and Multiple Health Outcomes: An Umbrella Review of Meta-Analyses
Source: Nutrients. 2023 Jul 25;15(15):3301. doi: 10.3390/nu15153301 (PMC10421296; doi:10.3390/nu15153301)
Supplement: Supplementary file 1 [file nutrients-15-03301-s001.zip › Table S2.pdf]

Supplementary Table S2. Summary of evidence classification for meta-analyses.

Evidence	Criteria
I, Consistent	• >1000 Cases<br>• $P<1\times10^{-6}$<br>• 95% prediction interval excludes null<br>• The largest original study reporting a statistically significant result ($P < 0.05$)<br>• No large heterogeneity ($I2 < 50\%$)<br>• No evidence of small-study effect ($P > 0.10$)<br>• No excess significance bias ($P > 0.10$)
II, Highly suggestive	• >1000 Cases<br>• $P<1\times10^{-6}$<br>• The largest original study reporting a statistically significant result ($P < 0.05$)
III, Suggestive	• >1000 Cases<br>• $p < 1\times10^{-3}$
IV, Weak	• Remaining $p < 0.05$
V, Not significant	• $p \geqslant 0.05$
