# Supplementary material for: Vitamin E and Multiple Health Outcomes: An Umbrella Review of Meta-Analyses
Source: Nutrients. 2023 Jul 25;15(15):3301. doi: 10.3390/nu15153301 (PMC10421296; doi:10.3390/nu15153301)
Supplement: Supplementary file 1 [file nutrients-15-03301-s001.zip › Table S3.pdf]

Supplementary Table S3. The list of excluded meta-analyses by full text screening with exclusion reason.

**Author, year**	**Reason for exclusion**
Xu R, et al. 2015[1]	Another meta-analysis studying the same association was chosen as the eligible meta-analysis
Cheng P, et al. 2018[2]	Another meta-analysis studying the same association was chosen as the eligible meta-analysis
Shrestha D S M, et al. 2013[3]	Another meta-analysis studying the same association was chosen as the eligible meta-analysis
Shang Y, et al. 2015[4]	Another meta-analysis studying the same association was chosen as the eligible meta-analysis
Schürks M, et al. 2010[5]	Another meta-analysis studying the same association was chosen as the eligible meta-analysis
Sato K, et al. 2015[6]	Another meta-analysis studying the same association was chosen as the eligible meta-analysis
Reyes R R P, et al. 2012[7]	Another meta-analysis studying the same association was chosen as the eligible meta-analysis
Saboori, et al. 2015[8]	Another meta-analysis studying the same association was chosen as the eligible meta-analysis
Miao H, et al. 2021[9]	Another meta-analysis studying the same association was chosen as the eligible meta-analysis
Li F J, et al. 2012[10]	Another meta-analysis studying the same association was chosen as the eligible meta-analysis
Ji H F, et al. 2014[11]	Another meta-analysis studying the same association was chosen as the eligible meta-analysis
Irani M, et al. 2020[12]	Another meta-analysis studying the same association was chosen as the eligible meta-analysis
Huang H, et al. 2016[13]	Another meta-analysis studying the same association was chosen as the eligible meta-analysis
Eum S, et al. 2013[14]	Another meta-analysis studying the same association was chosen as the eligible meta-analysis
Dong Y, et al. 2018[15]	Another meta-analysis studying the same association was chosen as the eligible meta-analysis
Dong R, et al. 2018[16]	Another meta-analysis studying the same association was chosen as the eligible meta-analysis
Cheng P, et al. 2018[17]	Another meta-analysis studying the same association was chosen as the eligible meta-analysis
Chen G, et al. 2015[18]	Another meta-analysis studying the same association was chosen as the eligible meta-analysis
Chen F, et al. 2015[19]	Another meta-analysis studying the same association was chosen as the eligible meta-analysis
Bin Q, et al. 2011[20]	Another meta-analysis studying the same association was chosen as the eligible meta-analysis
Amanullah I, et al. 2019[21]	Another meta-analysis studying the same association was chosen as the eligible meta-analysis
Alkhenizan A H, et al. 2004[22]	Another meta-analysis studying the same association was chosen as the eligible meta-analysis

Abdel-Maboud M, et al. 2020[23]	Another meta-analysis studying the same association was chosen as the eligible meta-analysis
Schürks M, et al. 2010[24]	Another meta-analysis studying the same association was chosen as the eligible meta-analysis
Zhang Z, et al. 2020[25]	Effects of Vitamin E and Combination with Other Substances on Health Outcomes
Zhang J, et al. 2021[26]	Effects of Vitamin E and Combination with Other Substances on Health Outcomes
Sepidarkish M, et al. 2019[27]	Effects of Vitamin E and Combination with Other Substances on Health Outcomes
Moosavian S P, et al. 2019[28]	Effects of Vitamin E and Combination with Other Substances on Health Outcomes
Jiang S, et al. 2014[29]	Effects of Vitamin E and Combination with Other Substances on Health Outcomes
Derakhshandeh S M, et al. 2020[30]	Effects of Vitamin E and Combination with Other Substances on Health Outcomes
Dehbalaei M G, et al. 2021[31]	Effects of Vitamin E and Combination with Other Substances on Health Outcomes
Chaitanya N C, et al. 2017[32]	Effects of Vitamin E and Combination with Other Substances on Health Outcomes
Bjelakovic G, et al. 2013[33]	Effects of Vitamin E and Combination with Other Substances on Health Outcomes
Asbaghi O, et al. 2020[34]	Effects of Vitamin E and Combination with Other Substances on Health Outcomes
Asbaghi O, et al. 2019[35]	Effects of Vitamin E and Combination with Other Substances on Health Outcomes
Yang S K, et al. 2014[36]	Not related to health outcomes
Li Z, et al. 2021[37]	Not related to health outcomes
Sahebkar A, et al. 2015[38]	Not related to health outcomes
Oliver C J, et al. 2017[39]	Not related to health outcomes
Moghimi-K M H, et al. 2020[40]	Not related to health outcomes
Huang J, et al. 2015[41]	Not related to health outcomes
Wyatt M C, et al. 2020[42]	Not related to health outcomes
D'Arrigo G, et al. 2017[43]	Not related to health outcomes
Curtis A J, et al. 2014[44]	Not related to health outcomes
Bourne N, et al. 2007[45]	Not related to health outcomes
Asleh R, et al. 2018[46]	Not related to health outcomes
Baggetta R, et al. 2016[47]	Not related to health outcomes
Yale S L, et al. 2005[48]	Not meta-analysis

Vardi M, et al. 2012[49]	Not meta-analysis
Trefan L, et al. 2011[50]	Not meta-analysis
Shelton R J, et al. 2005[51]	Not meta-analysis
Alkhenizan A, et al. 2007[52]	Provided insufficient data for quantitative synthesis
Barak Y, et al. 1998[53]	Provided insufficient data for quantitative synthesis
JianHai L, et al. 2019[54]	Full text could not be retrieved
Alcantara M J E, et al. 2020[55]	Full text could not be retrieved
Rad E Y, et al. 2018[56]	Full text could not be retrieved
Kumar, et al. 2013[57]	Another meta-analysis studying the same association was chosen as the eligible meta-analysis

References of the excluded meta-analyses by full text screening

[1] Xu R, Tao A, Zhang S, et al. Association between vitamin E and non-alcoholic steatohepatitis: a meta-analysis[J]. International Journal of Clinical and experimental medicine, 2015, 8(3): 3924.

[2] Cheng P, Wang L, Ning S, et al. Vitamin E intake and risk of stroke: a meta-analysis[J]. British Journal of Nutrition, 2018, 120(10): 1181-1188.

[3] Shrestha D S M, Rijal R, Shreshtha M, et al. Vitamin e in the treatment of NAFLD: Systematic review and metaanalysis[C]//JOURNAL OF GASTROENTEROLOGY AND HEPATOLOGY. 111 RIVER ST, HOBOKEN 07030-5774, NJ USA: WILEY-BLACKWELL, 2013, 28: 191-191.

[4] Shang Y, Yi S, Cui D, et al. Vitamin E Intake and Risk of Renal Cell Carcinoma: A Meta-Analysis of 7 Case – Control Studies[J]. Journal of Renal Nutrition, 2015, 25(4): 339-344.

[5] Schürks M, Glynn R J, Rist P M, et al. Effects of vitamin E on stroke subtypes: meta-analysis of randomised controlled trials[J]. Bmj, 2010, 341.

[6] Sato K, Gosho M, Yamamoto T, et al. Vitamin E has a beneficial effect on nonalcoholic fatty liver disease: a meta-analysis of randomized controlled trials[J]. Nutrition, 2015, 31(7-8): 923-930.

[7] Reyes R R P, Rhea Reyes R, Rhea Reyes R, et al. Efficacy of Vitamin E Supplementation in Cisplatin-Induced Neuropathy: A Meta-Analysis of Randomized Controlled Trials[C]//ANNALS OF NEUROLOGY. 111 RIVER ST, HOBOKEN 07030-5774, NJ USA: WILEY-BLACKWELL, 2012, 72: S36-S36.

[8] Saboori S, Shab-Bidar S, Speakman J R, et al. Effect of vitamin E supplementation on serum C-reactive protein level: a meta-analysis of randomized controlled trials[J]. European journal of clinical nutrition, 2015, 69(8): 867-873.

[9] Miao H, Li R, Chen D, et al. Protective effects of vitamin E on chemotherapy-induced peripheral neuropathy: a meta-analysis of randomized controlled trials[J]. Annals of Nutrition and Metabolism, 2021, 77(3): 127-137.

[10] Li F J, Shen L, Ji H F. Dietary intakes of vitamin E, vitamin C, and β-carotene and risk of Alzheimer's disease: a meta-analysis[J]. Journal of Alzheimer's disease, 2012, 31(2): 253-258.

[11] Ji H F, Sun Y, Shen L. Effect of vitamin E supplementation on aminotransferase levels in patients with NAFLD, NASH, and CHC: results from a meta-analysis[J]. Nutrition, 2014, 30(9): 986-991.

[12] Irani M, Sheidaei S, Ghazanfarpour M, et al. Comparative evaluation of evening primrose oil and vitamin E on the severity of cyclic Mastalgia: A Systematic Review and Meta-Analysis[J]. Iranian Journal of Obstetrics, Gynecology and Infertility, 2020, 23(3): 91-98.

[13] Huang H, He M, Liu L, et al. Vitamin E does not decrease the incidence of chemotherapy-induced peripheral neuropathy: a meta-analysis[J]. Contemporary Oncology, 2016, 20(3): 237.

[14] Eum S, Choi H D, Chang M J, et al. Protective effects of vitamin E on chemotherapy-induced peripheral neuropathy: a meta-analysis of randomized controlled trials[J]. Int J Vitam Nutr Res, 2013, 83(2): 101-111.

[15] Dong Y, Chen X, Liu Y, et al. Do low‐serum vitamin E levels increase the risk of Alzheimer disease in older people? Evidence from a meta‐analysis of case‐control studies[J]. International journal of geriatric psychiatry, 2018, 33(2): e257-e263.

[16] Dong R, Yang Q, Zhang Y, et al. Meta-analysis of vitamin C, vitamin E and β-carotene levels in the plasma of Alzheimer's disease patients[J]. Wei Sheng yan jiu= Journal of Hygiene Research, 2018, 47(4): 648-654.

[17] Cheng P, Wang L, Ning S, et al. Vitamin E intake and risk of stroke: a meta-analysis[J]. British Journal of Nutrition, 2018, 120(10): 1181-1188.

[18] Chen G, Wang J, Hong X, et al. Dietary vitamin E intake could reduce the risk of lung cancer: evidence from a meta-analysis[J]. International journal of clinical and experimental medicine, 2015, 8(4): 6631.

[19] Chen F, Li Q, Yu Y, et al. Association of vitamin C, vitamin D, vitamin E and risk of bladder cancer: a dose-response meta-analysis[J]. Scientific reports, 2015, 5(1): 1-7.

[20] Bin Q, Hu X, Cao Y, et al. The role of vitamin E (tocopherol) supplementation in the prevention of stroke[J]. Thrombosis and haemostasis, 2011, 105(04): 579-585.

[21] Amanullah I, Khan Y H, Anwar I, et al. Effect of vitamin E in non-alcoholic fatty liver disease: a systematic review and meta-analysis of randomised controlled trials[J]. Postgraduate Medical Journal, 2019, 95(1129): 601-611.

[22] Alkhenizan A H, Al-Omran M A. The role of vitamin E in the prevention of coronary events and stroke. Meta-analysis of randomized controlled trials[J]. Saudi medical journal, 2004, 25(12): 1808-1814.

[23] Abdel-Maboud M, Menshawy A, Menshawy E, et al. The efficacy of vitamin E in reducing non-alcoholic fatty liver disease: a systematic review, meta-analysis, and meta-regression[J]. Therapeutic advances in gastroenterology, 2020, 13: 1756284820974917.

[24] Schürks M, Glynn R J, Rist P M, et al. Effects of vitamin E on stroke subtypes: meta-analysis of randomised controlled trials[J]. Bmj, 2010, 341.

[25] Zhang Z, Xiao W, Jia J, et al. RETRACTED: the effect of combined application of pentoxifylline and vitamin E for the treatment of osteoradionecrosis of the jaws: a meta-analysis[J]. Oral Surgery, Oral Medicine, Oral Pathology and Oral Radiology, 2020, 129(3): 207-214.

[26] Zhang J, Xing C, Zhao H, et al. The effectiveness of coenzyme Q10, vitamin E, inositols, and vitamin D in improving the endocrine and metabolic profiles in women with polycystic ovary syndrome: a network Meta-analysis[J]. Gynecological Endocrinology, 2021, 37(12): 1063-1071.

[27] Sepidarkish M, Morvaridzadeh M, Akbari-Fakhrabadi M, et al. Effect of omega-3 fatty acid plus vitamin E Co-Supplementation on lipid profile: A systematic review and meta-analysis[J]. Diabetes & Metabolic Syndrome: Clinical Research & Reviews, 2019, 13(2): 1649-1656.

[28] Moosavian S P, Arab A, Mehrabani S, et al. The effect of omega-3 and vitamin E on oxidative stress and inflammation: Systematic review and meta-analysis of randomized controlled trials[J]. International Journal for Vitamin and Nutrition Research, 2019.

[29] Jiang S, Pan Z, Li H, et al. Meta-analysis: low-dose intake of vitamin E combined with other vitamins or minerals may decrease all-cause mortality[J]. Journal of nutritional science and vitaminology, 2014, 60(3): 194-205.

[30] Derakhshandeh-Rishehri S M, Heidari-Beni M, Eftekhari M H. THE EFFECTS OF REALSIL (SILYBIN – PHOSPHOLIPID – VITAMIN E COMPLEX) ON LIVER ENZYMES IN PATIENTS WITH NON-ALCOHOLIC FATTY LIVER DISEASE (NAFLD) OR NON-ALCOHOLIC STEATO-HEPATITIS (NASH): A SYSTEMATIC REVIEW AND META-ANALYSIS OF RCTS[J]. Acta Endocrinologica (Bucharest), 2020, 16(2): 223.

[31] Dehbalaei M G, Ashtary-Larky D, Mesrkanlou H A, et al. The effects of magnesium and vitamin E co-supplementation on some cardiovascular risk factors: A meta-analysis[J]. Clinical Nutrition ESPEN, 2021, 41: 110-117.

[32] Chaitanya N C, Muthukrishnan A, Babu D B G, et al. Role of vitamin E and vitamin a in oral mucositis induced by cancer chemo/radiotherapy-a meta-analysis[J]. Journal of clinical and diagnostic research: JCDR, 2017, 11(5): ZE06.

[33] Bjelakovic G, Nikolova D, Gluud C. Meta-regression analyses, meta-analyses, and trial sequential analyses of the effects of supplementation with beta-carotene, vitamin A, and vitamin E singly or in different combinations on all-cause mortality: do we have evidence for lack of harm?[J]. PloS one, 2013, 8(9): e74558.

[34] Asbaghi O, Saboori S, Falahi E, et al. The effect of omega-3 fatty acids and vitamin E co-supplementation on glycemic profile: A systematic review and meta-analysis of randomized controlled trials[J]. Obesity Medicine, 2020, 17: 100180.

[35] Asbaghi O, Choghakhori R, Abbasnezhad A. Effect of Omega-3 and vitamin E co-supplementation on serum lipids concentrations in overweight patients with metabolic disorders: A systematic review and meta-analysis of randomized controlled trials[J]. Diabetes & Metabolic Syndrome: Clinical Research & Reviews, 2019, 13(4): 2525-2531.

[36] Yang S K, Xiao L, Xu B, et al. Effects of vitamin E-coated dialyzer on oxidative stress and inflammation status in hemodialysis patients: a systematic review and meta-analysis[J]. Renal failure, 2014, 36(5): 722-731.

[37] Li Z, Xiang S, Wu C, et al. Vitamin E highly cross-linked polyethylene reduces mid-term wear in primary total hip replacement: a meta-analysis and systematic review of randomized clinical trials using radiostereometric analysis[J]. EFORT open reviews, 2021, 6(9): 759-770.

[38] Sahebkar A, Simental-Mendia L E, Ferretti G, et al. Statin therapy and plasma vitamin E concentrations: A systematic review and meta-analysis of randomized placebo-controlled trials[J]. Atherosclerosis, 2015, 243(2): 579-588.

[39] Oliver C J, Myers S P. Validity of a Cochrane Systematic Review and meta-analysis for determining the safety of vitamin E[J]. BMC complementary and alternative medicine, 2017, 17(1): 1-8.

[40] Moghimi-Kandelousi M H, Alamouti A A, Imani M, et al. A meta-analysis and meta-regression of the effects of vitamin E supplementation on serum enrichment, udder health, milk yield, and reproductive performance of transition cows[J]. Journal of dairy science, 2020, 103(7): 6157-6166.

[41] Huang J, Yi B, Li A, et al. Effects of vitamin E-coated dialysis membranes on anemia, nutrition and dyslipidemia status in hemodialysis patients: a meta-analysis[J]. Renal failure, 2015, 37(3): 398-407.

[42] Wyatt M C, Roberton A, Foxall-Smi M, et al. Does vitamin E highly-crosslinked polyethylene convey an advantage in primary total hip replacement? A systematic review and meta-analysis[J]. Hip International, 2020, 30(5): 598-608.

[43] D'Arrigo G, Baggetta R, Tripepi G, et al. Effects of vitamin E-coated versus conventional membranes in chronic hemodialysis patients: a systematic review and meta-analysis[J]. Blood Purification, 2017, 43(1-3): 101-122.

[44] Curtis A J, Bullen M, Piccenna L, et al. Vitamin E supplementation and mortality in healthy people: a meta-analysis of randomised controlled trials[J]. Cardiovascular drugs and therapy, 2014, 28(6): 563-573.

[45] Bourne N, Laven R, Wathes D C, et al. A meta-analysis of the effects of Vitamin E supplementation on the incidence of retained foetal membranes in dairy cows[J]. Theriogenology, 2007, 67(3): 494-501.

[46] Asleh R, Briasoulis A, Berinstein E M, et al. Meta-analysis of the association of the haptoglobin genotype with cardiovascular outcomes and the pharmacogenomic interactions with vitamin E supplementation[J]. Pharmacogenomics and personalized medicine, 2018, 11: 71.

[47] Baggetta R, D'Arrigo G, Galli F, et al. BENEFITS OF VITAMIN E-COATED VERSUS CONVENTIONAL MEMBRANES IN CHRONICHAEMODIALYSIS PATIENTS: A SYSTEMATIC REVIEW & META-ANALYSIS[C]//NEPHROLOGY DIALYSIS TRANSPLANTATION. GREAT CLARENDON ST, OXFORD OX2 6DP, ENGLAND: OXFORD UNIV PRESS, 2016, 31: 304-305.

[48] Yale S L. The Vitamin E Meta-Analysis: Cause for Concern?[J]. Alternative & Complementary Therapies, 2005, 11(1): 7-11.

[49] Vardi M, Blum S, Levy A P. Haptoglobin genotype and cardiovascular outcomes in diabetes mellitus—natural history of the disease and the effect of vitamin E treatment. Meta-analysis of the medical literature[J]. European Journal of Internal Medicine, 2012, 23(7): 628-632.

[50] Trefan L, Bünger L, Bloom-Hansen J, et al. Meta-analysis of the effects of dietary vitamin E supplementation on α-tocopherol concentration and lipid oxidation in pork[J]. Meat Science, 2011, 87(4): 305-314.

[51] Shelton R J, Velavan P, Nikitin N P, et al. Clinical trials update from the American Heart Association meeting: ACORN - CSD, primary care trial of chronic disease management, PEACE, CREATE, SHIELD, A - HeFT, GEMINI, vitamin E meta - analysis, ESCAPE, CARP, and SCD - HeFT cost - effectiveness study[J]. European journal of heart failure, 2005, 7(1): 127-135.

[52] Alkhenizan A, Hafez K. The role of vitamin E in the prevention of cancer: a meta-analysis of randomized controlled trials[J]. Annals of Saudi medicine, 2007, 27(6): 409-414.

[53] Barak Y, Swartz M, Shamir E, et al. Vitamin E (α-tocopherol) in the treatment of tardive dyskinesia: a statistical meta-analysis[J]. Annals of clinical psychiatry, 1998, 10(3): 101-105.

[54] JianHai L, ShaoJun C, Huan L, et al. Vitamin E consumption and the risk of bladder cancer: a meta-analysis of prospective studies[J]. International Journal for Vitamin and Nutrition Research, 2019, 89(3/4): 168-175.

[55] Alcantara M J E, Pandy J G P, Sebastian M R C. 338P Vitamin E in the treatment of chemotherapy and radiation-induced mucositis: A meta-analysis of randomized controlled trials[J]. Annals of Oncology, 2020, 31: S1372-S1373.

[56] Rad E Y, Falahi E, Djalali M, et al. Effect of vitamin E supplementation on plasma and urine levels of isoprostane F2 α in randomized controlled clinical trials: a systematic review and meta-analysis[J]. International Journal for Vitamin and Nutrition Research, 2018.

[57] Kumar S, Benjo A M, Pamidimukala C K, et al. Vitamin C decreases atrial fibrillation in cardiac surgery patients but vitamin e may decrease response: A randomized-controlled trial meta-analysis[J]. 2013.
