# Supplementary material for: Vitamin E and Multiple Health Outcomes: An Umbrella Review of Meta-Analyses
Source: Nutrients. 2023 Jul 25;15(15):3301. doi: 10.3390/nu15153301 (PMC10421296; doi:10.3390/nu15153301)
Supplement: Supplementary file 1 [file nutrients-15-03301-s001.zip › Table S4.pdf]

Supplementary Table S4. Assessments of AMSTAR scores.

Author	Year	AMSTAR 2 Items[a]																
		Domain																AMSTAR quality
		1	2	3	4	5	6	7	8	9	10	11	12	13	14	15	16	
Vadarlis	2021	Yes	Yes	No	Partial Yes	Yes	Yes	Yes	Yes	Yes	No	Yes	Yes	Yes	Yes	Yes	Yes	Moderate
Loh	2020	Yes	Yes	No	Partial Yes	Yes	Yes	Yes	Yes	Yes	No	Yes	Yes	Yes	Yes	Yes	Yes	Moderate
Nguyen	2021	Yes	Yes	No	Partial Yes	Yes	Yes	Yes	Yes	Yes	No	Yes	Yes	Yes	Yes	Yes	Yes	Moderate
Zhang	2015	Yes	Partial Yes	No	Partial Yes	Yes	Yes	Yes	Partial Yes	No	No	Yes	Yes	Yes	Yes	Yes	Yes	Low
Wang	2021	Yes	No	No	Partial Yes	Yes	Yes	Yes	Yes	Yes	No	Yes	Yes	Yes	Yes	Yes	Yes	Low
Hamedi	2021	Yes	No	No	Partial Yes	Yes	Yes	Yes	Yes	Yes	No	Yes	Yes	Yes	Yes	Yes	Yes	Low
Hajizadeh	2019	Yes	No	No	Partial Yes	Yes	Yes	Yes	Yes	Yes	No	Yes	Yes	Yes	Yes	Yes	Yes	Low
Emami	2021	Yes	No	No	Partial Yes	Yes	Yes	Yes	Yes	Yes	No	Yes	Yes	Yes	Yes	Yes	Yes	Low
Asbaghi	2020	Yes	No	No	Partial Yes	Yes	Yes	Yes	Yes	Yes	No	Yes	Yes	Yes	Yes	Yes	Yes	Low
Sarkhy	2014	Yes	No	No	Partial Yes	Yes	Yes	Yes	Yes	Yes	No	Yes	Yes	Yes	Yes	Yes	Yes	Low
Zhu	2017	Yes	No	Yes	Partial Yes	Yes	Yes	Yes	Yes	No	No	Yes	No	No	Yes	Yes	Yes	Critially Low
Xu	2014	Yes	No	No	Partial Yes	Yes	Yes	Yes	Yes	No	No	Yes	No	No	Yes	Yes	Yes	Critially Low
Wu	2018	Yes	No	No	Partial Yes	Yes	Yes	Yes	Yes	No	No	Yes	No	No	Yes	Yes	Yes	Critially Low
Stepanyan	2014	Yes	No	No	Partial Yes	Yes	Yes	Yes	Yes	Yes	Yes	Yes	Yes	Yes	Yes	No	Yes	Critially Low
Siervo	2015	Yes	Yes	No	Partial Yes	Yes	Yes	Yes	Yes	No	No	Yes	No	No	Yes	Yes	Yes	Critially Low
Shen	2015	Yes	No	No	Partial Yes	Yes	Yes	Partial Yes	Partial Yes	No	No	Yes	No	No	Yes	Yes	No	Critially Low
Qin	2014	Yes	No	No	Partial Yes	Yes	Yes	Yes	Partial Yes	No	No	Yes	No	No	Yes	Yes	Yes	Critially Low
Peng	2015	Yes	No	No	Partial Yes	Yes	Yes	Yes	Partial Yes	No	No	Yes	No	No	Yes	Yes	Yes	Critially Low
Mohammad	2021	Yes	No	No	Partial Yes	Yes	Yes	Yes	Yes	No	No	Yes	No	No	Yes	Yes	Yes	Critially Low
Loffredo	2015	Yes	No	No	Partial Yes	Yes	Yes	Partial Yes	Yes	No	No	Yes	No	No	Yes	Yes	Yes	Critially Low

Li	2016	Yes	No	No	Partial Yes	Yes	Yes	Yes	Partial Yes	No	No	Yes	No	No	Yes	Yes	Yes	Critially Low
Leng	2019	Yes	No	No	Partial Yes	Yes	Yes	Yes	Yes	No	No	Yes	No	No	Yes	Yes	Yes	Critially Low
Jayedi	2019	Yes	No	No	Partial Yes	Yes	Yes	Yes	Partial Yes	No	No	Yes	No	No	Yes	Yes	Yes	Critially Low
Hu	2017	Yes	Yes	No	Partial Yes	Yes	Yes	Yes	Partial Yes	No	No	Yes	No	No	Yes	Yes	Yes	Critially Low
Etminan	2005	Yes	No	No	Partial Yes	Yes	Yes	Partial Yes	Partial Yes	No	No	Yes	No	No	Yes	Yes	Yes	Critially Low
Emami	2019	Yes	No	No	Partial Yes	Yes	Yes	Yes	Yes	No	No	Yes	No	No	Yes	Yes	Yes	Critially Low
Dong	2017	Yes	No	No	Partial Yes	Yes	Yes	Yes	Yes	No	No	Yes	No	No	Yes	Yes	Yes	Critially Low
Cui	2018	Yes	No	No	Partial Yes	Yes	Yes	Yes	Partial Yes	No	No	Yes	No	No	Yes	Yes	Yes	Critially Low
Cho	2017	Yes	No	No	Partial Yes	Yes	Yes	Yes	Yes	No	No	Yes	No	No	Yes	Yes	Yes	Critially Low
Chen	2021	Yes	No	No	Partial Yes	Yes	Yes	Yes	Yes	No	No	Yes	No	No	Yes	Yes	Yes	Critially Low
Chen	2015	Yes	No	No	Partial Yes	Yes	Yes	Yes	Yes	No	No	Yes	No	Yes	Yes	Yes	Yes	Critially Low
Cui	2014	Yes	No	No	Partial Yes	Yes	Yes	Partial Yes	Yes	No	No	Yes	No	No	Yes	Yes	Yes	Critially Low

a. The revised AMSTAR (AMSTAR 2) has 13 items for systematic reviews and 16 items for meta-analyses.

1 – Did the research questions and inclusion criteria for the review include the components of PICO? (yes/no)

2 – Did the report of the review contain an explicit statement that the review methods were established prior to the conduct of the review and did the report justify any significant deviations from the protocol? (yes/partial yes/no)

3 – Did the review authors explain their selection of the study designs for inclusion in the review? (yes/no)

4 – Did the review authors use a comprehensive literature search strategy? (yes/partial yes/no)

5 – Did the review authors perform study selection in duplicate? (yes/no)

6 – Did the review authors perform data extraction in duplicate? (yes/no)

7 – Did the review authors provide a list of excluded studies and justify the exclusions? (yes/partial yes/no)

8 – Did the review authors describe the included studies in adequate detail? (yes/partial yes/no)

9 – Did the review authors use a satisfactory technique for assessing the risk of bias (RoB) in individual studies that were included in the review? (yes/partial yes/no)

10 – Did the review authors report on the sources of funding for the studies included in the review? (yes/no)

11 – If meta-analysis was performed, did the review authors use appropriate methods for statistical combination of results? (yes/no/no meta-analysis conducted)

12 – If meta-analysis was performed, did the review authors assess the potential impact of RoB in individual studies on the results of the meta-analysis or other evidence synthesis? (yes/no/no meta-analysis conducted)

13 – Did the review authors account for RoB in individual studies when interpreting/discussing the results of the review? (yes/no)

14 – Did the review authors provide a satisfactory explanation for, and discussion of, any heterogeneity observed in the results of the review? (yes/no)

15 – If they performed quantitative synthesis, did the review authors carry out an adequate investigation of publication bias (small study bias) and discuss its likely impact on the results of the review? (yes/no/no meta-analysis conducted)

16 – Did the review authors report any potential sources of conflict of interest, including any funding they received for conducting the review? (yes/no)
