# Supplementary material for: Vitamin E and Multiple Health Outcomes: An Umbrella Review of Meta-Analyses
Source: Nutrients. 2023 Jul 25;15(15):3301. doi: 10.3390/nu15153301 (PMC10421296; doi:10.3390/nu15153301)
Supplement: Supplementary file 1 [file nutrients-15-03301-s001.zip › Table S5.pdf]

Supplementary Table S5. GRADE classification of quality of evidence of all included meta-analyses.

Outcome	Author	Year	Type of included studies	Risk of bias	Inconsistency	Indirectness	Imprecision	Publication bias	Magnitude of effect	Plausible confounding	Dose-response	Quality
**Supplemental vitamin E**												
Adult patients with non-alcoholic fatty liver disease (NAFLD): alanine aminotransferase	Vadarlis	2021	RCT	Serious	Not serious	Not serious	Very serious	Undetected	No	No	No	⊕◯◯◯ Very low
Adult patients with non-alcoholic fatty liver disease (NAFLD): aspartate aminotransferase	Vadarlis	2021	RCT	Serious	Not serious	Not serious	Very serious	Strongly suspected	No	No	No	⊕◯◯◯ Very low
Age-related cataract	Zhang	2015	Cohort, RCT, Case–control	Serious	Very serious	Not serious	Serious	Undetected	No	No	No	⊕◯◯◯ Very low
ApoA	Hamedi	2021	RCT	Serious	Very serious	Not serious	Very serious	Undetected	No	No	No	⊕◯◯◯ Very low
ApoB	Hamedi	2021	RCT	Serious	Very serious	Not serious	Very serious	Undetected	No	No	No	⊕◯◯◯ Very low
Asthma or wheeze in children	Wu	2018	Case–control, Cross-sectional	Serious	Not serious	Not serious	Serious	Strongly suspected	No	No	No	⊕◯◯◯ Very low
Asthma or wheeze in children	Wu	2018	Cohort	Serious	Very serious	Not serious	Serious	Strongly suspected	No	No	No	⊕◯◯◯ Very low
Asthma or wheeze in children	Wu	2018	Cohort	Serious	Very serious	Not serious	Serious	Undetected	No	No	No	⊕◯◯◯ Very low
Bladder cancer	Chen	2015	Cohort, Case–control	Serious	Not serious	Not serious	Serious	Undetected	No	No	Yes	⊕⊕⊕◯ Moderate
BMI	Emami	2021	RCT	Serious	Not serious	Not serious	Very serious	Undetected	No	No	Yes	⊕⊕◯◯ Low
Cardiovascular mortality	Jayedi	2019	Cohort	Serious	Very serious	Not serious	Serious	Undetected	No	No	Yes	⊕◯◯◯

												Very low
Chemotherapy-induced peripheral neuropathy (CIPN)	Chen	2021	RCT	Serious	Very serious	Not serious	Very serious	Strongly suspected	No	No	No	⊕◯◯◯ Very low
Contrast-induced acute kidney injury	Cho	2017	RCT	Serious	Not serious	Not serious	Very serious	Strongly suspected	No	No	No	⊕◯◯◯ Very low
Diastolic blood pressure	Emami	2019	RCT	Serious	Very serious	Not serious	Very serious	Undetected	No	No	Yes	⊕◯◯◯ Very low
Duration of mastalgia	Hajizadeh	2019	RCT	Serious	Not serious	Not serious	Very serious	Undetected	No	No	No	⊕◯◯◯ Very low
Endothelial function	Siervo	2015	RCT	Serious	Very serious	Not serious	Serious	Undetected	No	No	Yes	⊕◯◯◯ Very low
Fasting glucose	Xu	2014	RCT	Serious	Very serious	Not serious	Very serious	Undetected	No	No	No	⊕◯◯◯ Very low
Fasting insulin	Xu	2014	RCT	Serious	Very serious	Not serious	Very serious	Undetected	No	No	No	⊕◯◯◯ Very low
Fatal myocardial infarction	Loffredo	2015	RCT	Serious	Not serious	Not serious	Not serious	Strongly suspected	No	No	No	⊕⊕◯◯ Low
HbA1c	Xu	2014	RCT	Serious	Very serious	Not serious	Very serious	Undetected	No	No	No	⊕◯◯◯ Very low
High-density lipoprotein cholesterol (HDL-C) levels in patients with diabetes mellitus	Mohammad	2021	RCT	Serious	Very serious	Not serious	Very serious	Undetected	No	No	No	⊕◯◯◯ Very low
Lipid peroxidation(malondialdehyde)	Stepanyan	2014	RCT	Serious	Serious	Not serious	Very serious	Undetected	No	No	No	⊕◯◯◯ Very low
Low-density lipoprotein cholesterol (LDL- C) levels in patients with diabetes mellitus	Mohammad	2021	RCT	Serious	Very serious	Not serious	Very serious	Undetected	No	No	No	⊕◯◯◯ Very low
Mean arterial pressure	Emami	2019	RCT	Serious	Very serious	Not serious	Very serious	Undetected	No	No	No	⊕◯◯◯ Very low
Muscle damage(creatine	Stepanyan	2014	RCT	Serious	Serious	Not serious	Very serious	Undetected	No	No	No	⊕◯◯◯

kinase)													Very low
Myocardial infarction	Loffredo	2015	RCT	Serious	Very serious	Not serious	Not serious	Strongly suspected	No	No	No	⊕◯◯◯ Very low
Non fatal myocardial infarction	Loffredo	2015	RCT	Serious	Very serious	Not serious	Serious	Undetected	No	No	No	⊕◯◯◯ Very low
Ovarian cancer	Leng	2019	Cohort, Case–control	Serious	Very serious	Not serious	Serious	Undetected	No	No	No	⊕◯◯◯ Very low
Patients Receiving Hemodialysis: Biomarkers of Endothelial Dysfunction-ICAM-1	Nguyen	2021	RCT	Serious	Very serious	Not serious	Very serious	Undetected	No	No	No	⊕◯◯◯ Very low
Patients Receiving Hemodialysis: Biomarkers of Endothelial Dysfunction-VCAM-1	Nguyen	2021	RCT	Serious	Very serious	Not serious	Very serious	Undetected	No	No	No	⊕◯◯◯ Very low
Patients Receiving Hemodialysis: Biomarkers of inflammation-CRP	Nguyen	2021	RCT	Serious	Very serious	Not serious	Very serious	Undetected	No	No	No	⊕◯◯◯ Very low
Patients Receiving Hemodialysis: Biomarkers of inflammation-IL-6	Nguyen	2021	RCT	Serious	Serious	Not serious	Very serious	Undetected	No	No	No	⊕◯◯◯ Very low
Patients Receiving Hemodialysis: Biomarkers of Oxidative Stress-malondialdehyde (MDA)	Nguyen	2021	RCT	Serious	Very serious	Not serious	Very serious	Strongly suspected	No	No	No	⊕◯◯◯ Very low
Pediatric Nonalcoholic Fatty Liver Disease	Sarkhy	2014	RCT	Serious	Not serious	Not serious	Serious	Undetected	No	No	No	⊕⊕◯◯ Low
Risk of Alzheimer's disease (AD)	Wang	2021	Cohort	Serious	Very serious	Not serious	Serious	Undetected	No	No	No	⊕◯◯◯ Very low
Serum CRP concentrations	Asbaghi	2020	RCT	Serious	Very serious	Not serious	Very serious	Undetected	No	No	No	⊕◯◯◯ Very low

Serum IL-6 concentrations	Asbaghi	2020	RCT	Serious	Very serious	Not serious	Very serious	Undetected	No	No	No	⊕◯◯◯ Very low
Serum TNF-α concentrations	Asbaghi	2020	RCT	Serious	Very serious	Not serious	Very serious	Undetected	No	No	Yes	⊕◯◯◯ Very low
Severity of mastalgia	Hajizadeh	2019	RCT	Serious	Very serious	Not serious	Very serious	Undetected	No	No	No	⊕◯◯◯ Very low
Stroke	Loh	2020	RCT	Serious	Not serious	Not serious	Serious	Undetected	No	No	No	⊕⊕◯◯ Low
Systolic blood pressure	Emami	2019	RCT	Serious	Very serious	Not serious	Very serious	Undetected	No	No	No	⊕◯◯◯ Very low
Total cholesterol (TC) levels in patients with diabetes mellitus	Mohammad	2021	RCT	Serious	Very serious	Not serious	Very serious	Undetected	No	No	No	⊕◯◯◯ Very low
Triglyceride (TG) levels in patients with diabetes mellitus	Mohammad	2021	RCT	Serious	Very serious	Not serious	Very serious	Undetected	No	No	No	⊕◯◯◯ Very low
Waist circumference (WC)	Emami	2021	RCT	Serious	Not serious	Not serious	Very serious	Strongly suspected	No	No	Yes	⊕◯◯◯ Very low
Weight	Emami	2021	RCT	Serious	Not serious	Not serious	Very serious	Undetected	No	No	Yes	⊕⊕◯◯ Low
**Dietary vitamin E**												
Age-related cataract	Zhang	2015	Cohort, Case–control, Cross-sectional	Serious	Very serious	Not serious	Serious	Undetected	No	No	Yes	⊕◯◯◯ Very low
Bladder cancer	Chen	2015	Cohort, Case–control	Serious	Not serious	Not serious	Serious	Strongly suspected	No	No	Yes	⊕⊕◯◯ Low
Cervical cancer	Hu	2017	Case–control	Serious	Very serious	Not serious	Not serious	Undetected	No	No	No	⊕◯◯◯ Very low
CIN, cervical intraepithelial neoplasia	Hu	2017	Case–control	Serious	Very serious	Not serious	Not serious	Undetected	No	No	No	⊕◯◯◯ Very low
Esophageal Cancer	Cui	2018	Cohort, Case–control	Serious	Very serious	Not serious	Serious	Strongly suspected	No	No	Yes	⊕◯◯◯ Very low

Kidney Cancer	Shen	2015	Cohort, Case–control	Serious	Very serious	Not serious	Serious	Undetected	No	No	No	⊕○○○ Very low
Lung cancer	Zhu	2017	Cohort	Serious	Very serious	Not serious	Serious	Undetected	No	No	Yes	⊕○○○ Very low
Ovarian cancer	Leng	2019	Cohort, Case–control	Serious	Very serious	Not serious	Serious	Undetected	No	No	No	⊕○○○ Very low
Parkinson's disease	Etminan	2005	Cohort, Case–control, Cross-sectional	Serious	Not serious	Not serious	Very serious	Undetected	No	No	No	⊕○○○ Very low
**Total vitamin E**												
Age-related cataract	Zhang	2015	Cohort, Case–control	Serious	Serious	Not serious	Serious	Undetected	No	No	No	⊕○○○ Very low
Bladder cancer	Chen	2015	Cohort, Case–control	Serious	Serious	Not serious	Very serious	Undetected	No	No	No	⊕○○○ Very low
Glioma	Qin	2014	Cohort, Case–control	Serious	Very serious	Not serious	Very serious	Undetected	No	No	No	⊕○○○ Very low
Ovarian cancer	Leng	2019	Cohort, Case–control	Serious	Very serious	Not serious	Serious	Undetected	No	No	No	⊕○○○ Very low
Pancreatic Cancer	Peng	2015	Cohort, Case–control	Serious	Not serious	Not serious	Serious	Strongly suspected	No	No	No	⊕○○○ Very low
**Circulating *α*-tocopherol levels**												
Age-related cataract	Zhang	2015	Cohort, Case–control, Cross-sectional	Serious	Very serious	Not serious	Serious	Strongly suspected	No	No	No	⊕○○○ Very low
Asthma or wheeze in children	Wu	2018	Cohort	Serious	Not serious	Not serious	Serious	Strongly suspected	No	No	No	⊕○○○ Very low
Bladder cancer	Chen	2015	Cohort, Case–control	Serious	Not serious	Not serious	Very serious	Undetected	No	No	Yes	⊕⊕○○ Low
Cardiovascular mortality	Jayedi	2019	Cohort	Serious	Not serious	Not serious	Very serious	Undetected	No	No	No	⊕○○○

												Very low
Colorectal cancer	Dong	2017	Case–control	Serious	Very serious	Not serious	Very serious	Undetected	No	No	No	⊕◯◯◯ Very low
CVD	Li	2016	Cohort, Case–control	Serious	Very serious	Not serious	Very serious	Undetected	No	No	No	⊕◯◯◯ Very low
Prostate cancer	Cui	2014	Case–control	Serious	Not serious	Not serious	Very serious	Strongly suspected	No	No	Yes	⊕◯◯◯ Very low
